# High intensity aerobic exercise training improves chronic intermittent hypoxia-induced insulin resistance without basal autophagy modulation

**DOI:** 10.1038/srep43663

**Published:** 2017-03-03

**Authors:** Marion Pauly, Allan Assense, Aurélie Rondon, Amandine Thomas, Hervé Dubouchaud, Damien Freyssenet, Henri Benoit, Josiane Castells, Patrice Flore

**Affiliations:** 1 Grenoble Alpes University, HP2 laboratory, Grenoble, F-38042, France; 2 INSERM, U1042, Grenoble, F-38042, France; 3 INSERM, U1055, LBFA, Grenoble Alpes University, Grenoble, France; 4 Université Lyon, Université Jean Monnet Saint Etienne, Laboratoire Interuniversitaire de Biologie de la Motricité, EA7424, F-42023, Saint Etienne, France

## Abstract

Chronic intermittent hypoxia (IH) associated with obstructive sleep apnea (OSA) is a major risk factor for cardiovascular and metabolic diseases (insulin resistance: IR). Autophagy is involved in the pathophysiology of IR and high intensity training (HIT) has recently emerged as a potential therapy. We aimed to confirm IH-induced IR in a tissue-dependent way and to explore the preventive effect of HIT on IR-induced by IH. Thirty Swiss 129 male mice were randomly assigned to Normoxia (N), Intermittent Hypoxia (IH: 21–5% FiO_2_, 30 s cycle, 8 h/day) or IH associated with high intensity training (IH HIT). After 8 days of HIT (2*24 min, 50 to 90% of Maximal Aerobic Speed or MAS on a treadmill) mice underwent 14 days IH or N. We found that IH induced IR, characterized by a greater glycemia, an impaired insulin sensitivity and lower AKT phosphorylation in adipose tissue and liver. Nevertheless, MAS and AKT phosphorylation were greater in muscle after IH. IH associated with HIT induced better systemic insulin sensitivity and AKT phosphorylation in liver. Autophagy markers were not altered in both conditions. These findings suggest that HIT could represent a preventive strategy to limit IH-induced IR without change of basal autophagy.

Obstructive sleep apnea (OSA) syndrome is characterized by recurrent episodes of pharyngeal collapses occurring during sleep, leading to chronic intermittent hypoxia (IH). OSA is well established as a cardiovascular risk factor^1^. Insulin resistance (IR) and diabetes are two cardiovascular risk factors promoted by OSA^1^. Indeed, OSA is associated with an increased prevalence of type 2 diabetes and has been shown to be a risk factor for insulin resistance and incident diabetes^2^,^3^,^4^.

Clinical and experimental studies have shown the major role of chronic IH in the insulin resistance induced by OSA. Furthermore, IR induced by IH can occur independently of obesity^1^. Several factors in OSA/IH patients may lead to IR among which: sympathetic nervous system hyper-activity, decrease in insulin growth factor I (IGF-1), hyperlipidemia induced proinflammatory and oxidative stress pathways (NF-ĸB pathway, reactive oxygen species), ectopic fat and release of inflammatory cytokines^1^. Muscle is particularly important in the regulation of glucose metabolism since it can account for 80% of glucose disposal^5^. Intermittent hypoxia induced IR has been shown in the soleus muscle of mice independently of autonomic activity^6^. Mechanisms involved in this IR are not fully understood and deserve attention.

The first-line therapy for OSA is the application of continuous positive airway pressure (CPAP), which substantially decreases the number and severity of respiratory events in patients^7^. Its efficiency on IR is not clear^8^ however slight improvement of insulin resistance has been observed after 2 weeks of 8 h of nightly treatment, a compliance scarcely reached by patients^9^. Thus, a better understanding of the pathophysiological mechanisms involved in the deleterious effects of IH could help to provide new alternative or complementary treatment to CPAP in the management of OSA-associated metabolic impairment.

The role of autophagy in metabolic alterations and, in particular IR, has been well documented^10^. Autophagy consists of a cellular recycling program that controls the quality of cellular elements by turning over cytoplasmic components within lysosomes. It protects against cellular toxicity and death by recycling oxidized proteins and aged organelles. It is finely regulated by more than 30 autophagy related genes and involves several steps after induction: generation of the nucleation complex, autophagosome formation, and cargo recognition^11^. Nucleation complex requires the dissociation of Beclin1 from Bcl-2, which leads to the functional class III phosphatidylinositol 3-kinase (PI3K) complex facilitating the targeting of other autophagic molecules to the nucleation complex. Thereafter, recruitment of membranes to the nucleation complex promotes formation of the limiting membrane^12^ Finally, membrane elongation and autophagosome formation occurs following activation of Atg5-Atg12 and the microtubule-associated protein light chain 3 (LC3), which may be lipidated to membrane-associated phosphatidylethanolamine^10^. Sealing upon itself, the limiting membrane can sequester cargo within double-membraned autophagosomes. Then cargo can be hydrolyzed after fusion of autophagosomes with lysosomes.

Numerous signaling factors converge to the metabolic sensor mammalian target of rapamycin mTOR to fine-tune autophagy. Briefly when nutrients and growth factors are available, mTOR inhibits autophagy. By contrast, energy depletion reduces the inhibitory effects of mTOR on autophagy through an increase of AMPK activity. Moreover, autophagy can be inhibited by the insulin pathway PI3K-AKT-mTOR and mTOR activation is regulated by AKT^13^. On a cellular model, hypoxia itself, via the transcription factor HIF-1, directly activates autophagy through BNIP3 regulation (allowing dissociation of Beclin from Bcl-2)^14^.

Moreover, autophagy has been shown to be activated in situations of energy imbalance such as stress, starvation and exercise^15^. However, its regulation seems more complex and tissue dependent. Exercise training activates autophagy in muscle to overcome insulin resistance developed in the case of overnutrition^16^. Others factors can negatively influence autophagy among which insulin^17^, high fat feeding, lipid toxicity and the resulting IR, hepatic steatosis and inflammation^10^. Previous studies have shown that livers from dietary and genetic mouse models of obesity decreased autophagy, resulting in insulin resistance^18^. Conversely, obesity might be associated with increased autophagy in adipose tissue, leading to an increase in circulating lipids^19^.

The question whether the insulin resistance associated to OSA or IH is related to modulation of autophagy in a tissue dependent manner remains unanswered. To our knowledge, apart from a study showing the importance of autophagy to maintain cardiac contractility^20^, the effects of chronic IH on autophagy and its relationship with insulin resistance has never been studied. We hypothesized that chronic IH exposure would fail to activate autophagy, in a tissue dependent manner, due to the alteration of insulin sensitivity in OSA.

Beneficial effects of aerobic exercise on metabolic health and particularly insulin resistance are well established^21^. Intense exercise intensity (high intensity training: HIT) seems suitable and more efficient to get rapid benefits on metabolic health on whole body insulin sensitivity and some of its contributors such muscle microvascular density and eNOS protein^22^,^23^. In previous studies performed on rat^24^ and in mice (unpublished results), we assessed metabolic and functional variables to determine which protocol (moderate intensity aerobic endurance training or high-intensity aerobic endurance training (HIT)) of equal volume (i.e. total energy expenditure although different intensity) promoted more beneficial outcomes after short-term training. We selected the HIT used in the present study since only 10 sessions elicited the greater effects regarding training performance (i.e. maximal aerobic speed and endurance) and muscle adaptations (i.e. citrate synthase activity).

Thus, our second hypothesis was that HIT would reduce insulin resistance and improve autophagy status in a context of intermittent hypoxia.

If our hypotheses are verified, targeting IH induced IR by HIT could represent a new promising preventive strategy in OSA patients.

## Results

### Effect of intermittent hypoxia associated or not with high intensity exercise training on weight, food intake, hematocrit and exercise performance

**Figure 1:**
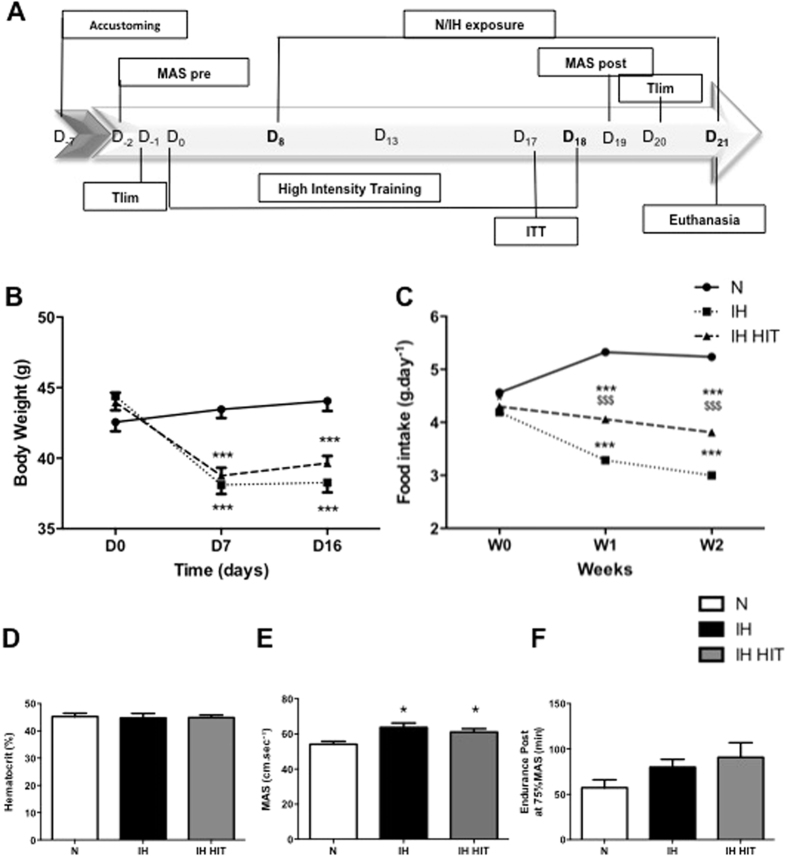
Effect of intermittent hypoxia with or without high intensity exercise training on body weight, hematocrit, food intake and exercise performance. (**A**) Experimental design of the study. After 7 days of habituation to the treadmill, animals started 18 days of High Intensity Training on a 0% incline. Intermittent Hypoxia began at day 8 of HIT for 14 days. 3 days before euthanasia, insulin tolerance test, maximal aerobic speed and endurance tests have been performed. Mice weight evolution (**B**) and food intake (**C**) over the exposure to IH and HIT. Running performance: (**D**) Maximal Aerobic Speed (MAS) and (**E**) endurance were measured. (**F**) Hematocrit levels were measured on mice capillaries collected at D_17_ during the Insulin Tolerance Test. Data are presented as mean ± SEM. *P < 0.05, ***P < 0.001 vs N, ^$$$^P < 0.001 IH HIT vs IH. (n = 9–10 per group).

The experimental design is described in Fig. 1A. Intermittent hypoxia, independently of exercise training, decreased weight after 7 days of exposure (N: 43.4 ± 0.6 g; IH: 38.1 ± 0.7 g; IH HIT: 38.7 ± 0.6 g; p < 0.001). Thereafter the weight plateaued until the last day of exposure (Fig. 1B). This weight loss was paralleled by a decrease in food intake in both groups exposed to intermittent hypoxia (N: 5.2 ± 5.2; IH: 3.0 ± 0.1; IH HIT: 3.8 ± 0.1 g.day^−1^; p < 0.001; Fig. 1C). However, exercise training induced a greater food intake compared to the non-trained group, which prevented further weight loss in this group (Fig. 1C). Hematocrit did not differ between the 3 groups (Fig. 1D). Post IH exposition, maximal aerobic speed (MAS) was greater after hypoxia compared to normoxia whether associated or not to exercise training (Fig. 1E). Endurance at 75% of MAS followed the same tendency but did not reach statistical significance (p = 0,095; Fig. 1F).

### Impact of intermittent hypoxia on insulin sensitivity

**Figure 2:**
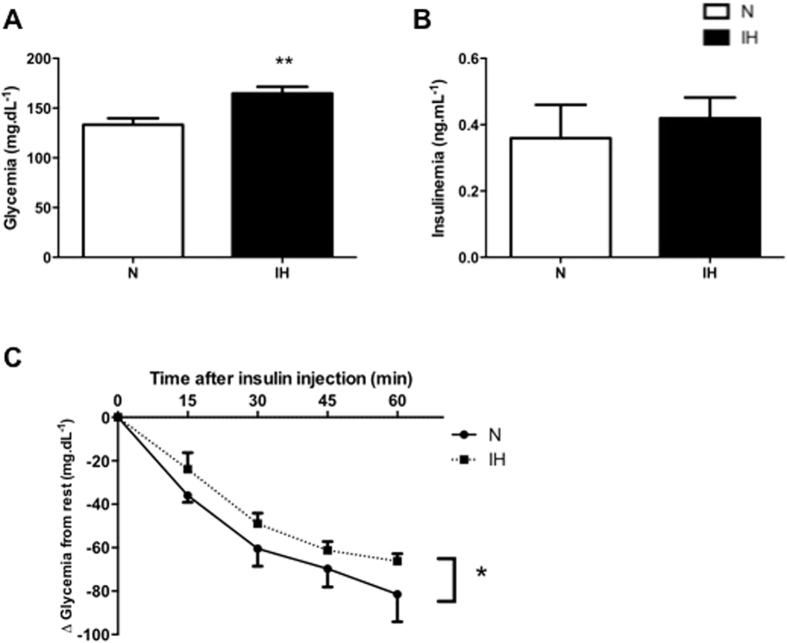
Impact of intermittent hypoxia on insulin sensitivity. Mice fasted glycemia (**A**), Insulinemia (**B**) and Insulin Tolerance Test (ITT) (**C**) after intermittent hypoxia exposure. Blood glucose or plasma insulin was measured before intraperitoneally injection of insulin (T0). Data are presented as mean ± SEM. *P < 0.05, **P < 0.005 vs N (n = 9–10 per group).

Fasting glycemia was significantly higher after intermittent hypoxia compared to normoxia (N: 133.3 ± 6.5 mg.dL^−1^; IH: 164.7 ± 6.7 mg.dL^−1^; p < 0.01; Fig. 2A) whereas fasting insulinemia did not significantly differ (Fig. 2B). Glycemia measured during 90 minutes after insulin injection was less decreased after intermittent hypoxia compared to normoxia, indicating insulin resistance (F = 4.183; P = 0.044; Fig. 2C).

### Impact of intermittent hypoxia on insulin and autophagy activation signaling pathways

**Figure 3:**
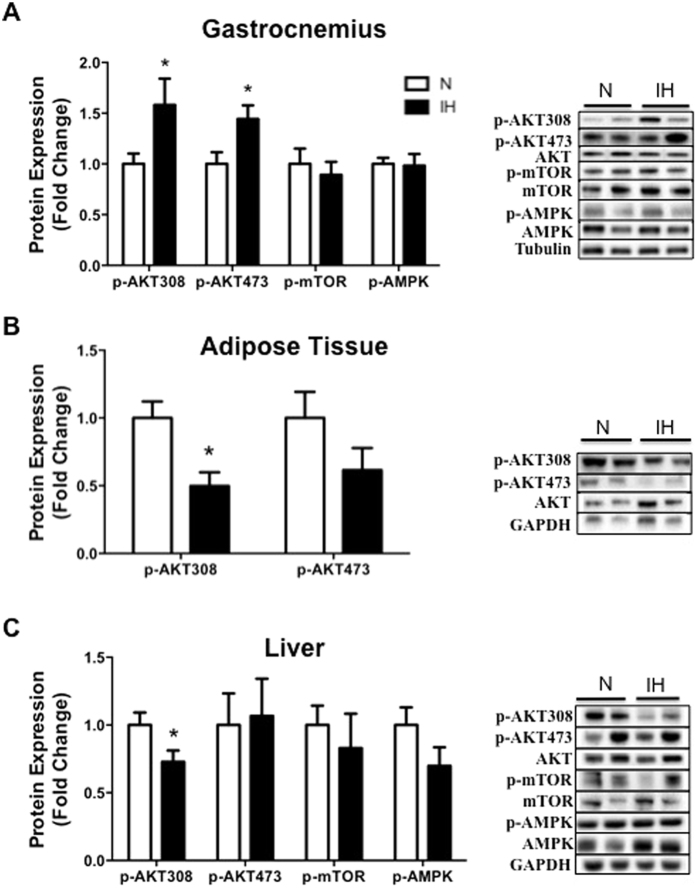
Impact of intermittent hypoxia on insulin and autophagy activation signaling pathways in muscle, adipose tissue and liver. Quantification of phosphorylation of AKT (Thr308 and Ser473), mTOR (Ser2448) and AMPK (Th172) protein expression and representative immunoblot images in (**A**) gastrocnemius muscle, (**B**) adipose tissue and (**C**) liver. AMPK and mTOR were undetectable in adipose tissue. Comparisons were performed after normalization to the total form of the protein. Data are presented as mean ± SEM. *P < 0.05 vs N (n = 9–10 per group).

In *gastrocnemius*, AKT phosphorylations on serine 308 (Fig. 3A; 1.6 ± 0.3 fold increase; p < 0.05) and on threonine 473 (Fig. 3A; 1.4 ± 0.1 fold control value; p < 0.05) were greater after intermittent hypoxia compared to normoxia whereas neither AMPK (Fig. 3A) nor mTOR phosphorylations differed between the 2 groups (Fig. 3A). Conversely in *adipose tissue*, AKT phosphorylations on serine 308 (Fig. 3B; p < 0.05) and on threonine 473 (Fig. 3B; p < 0.15) were lower after intermittent hypoxia although for the latter it did not reach statistical significance. In the *liver*, only AKT phosphorylation on serine 308 (Fig. 3C; 0.7 ± 0.1 fold control value; p < 0.05) was significantly lower compared to normoxia; AKT phosphorylation on threonine 473, AMPK and mTOR did not differ (Fig. 3C).

### Impact of intermittent hypoxia on autophagy markers

**Figure 4:**
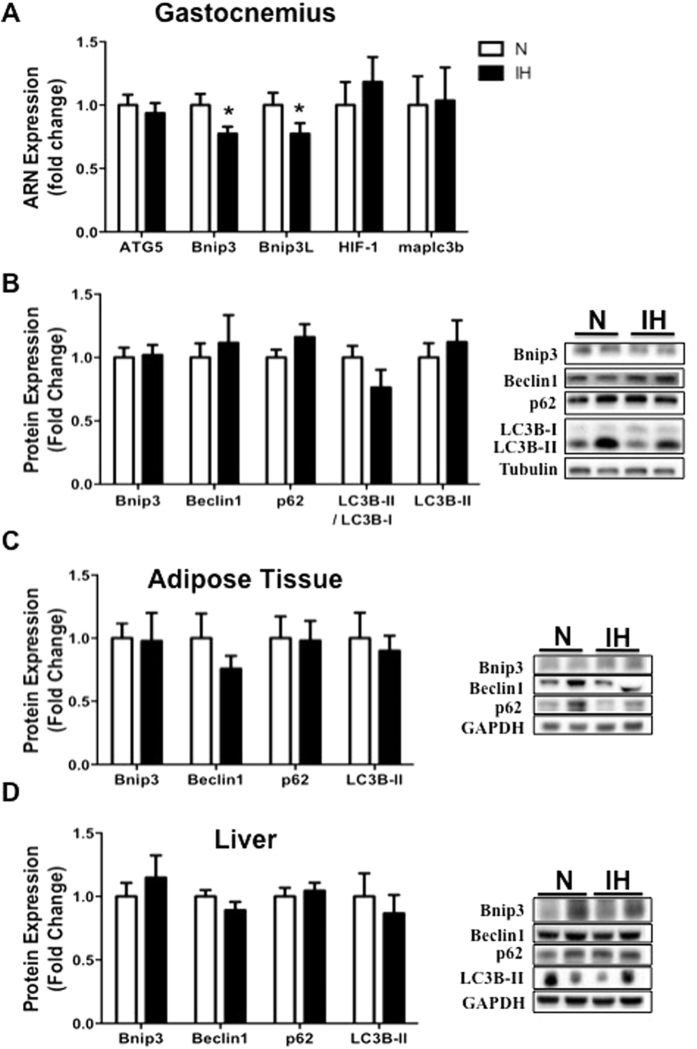
Impact of intermittent hypoxia on autophagy markers in muscle, adipose tissue and liver. mRNA (**A**) and protein (**B**–**D**) analyses were performed on mice that underwent normoxia (N) or fourteen days of intermittent hypoxia (IH). Quantification of phosphorylation of Bnip3, Beclin1, p62, the ratio LC3-II/LC3-I protein expression and representative immunoblot images in (**B**) gastrocnemius muscle, (**C**) adipose tissue and (**D**) liver. Comparisons were performed after normalization to α-Tubulin or GAPDH. Data are presented as mean ± SEM. *P < 0.05 vs N(n = 9–10 per group).

In *gastrocnemius*, autophagy protein expressions such Bnip3, Beclin1, p62 and LC3B-II/LC3B-I did not differ between IH and N (Fig. 4A). Bnip3 (0.8 ± 0.1 fold control value; p < 0.01) and Bnip3L (0.8 ± 0.1 fold control value; p < 0.05) mRNA relative expressions were lower after IH compared to N whereas those of ATG5 or LC3B were similar (Fig. 4B). In *adipose tissue*, none of measured protein expression among which Bnip3, Beclin1, p62, LC3B differed between IH and N (Fig. 4C). The same applied in *liver* (Fig. 4D).

### Effects of high intensity training on intermittent hypoxia induced insulin resistance

**Figure 5:**
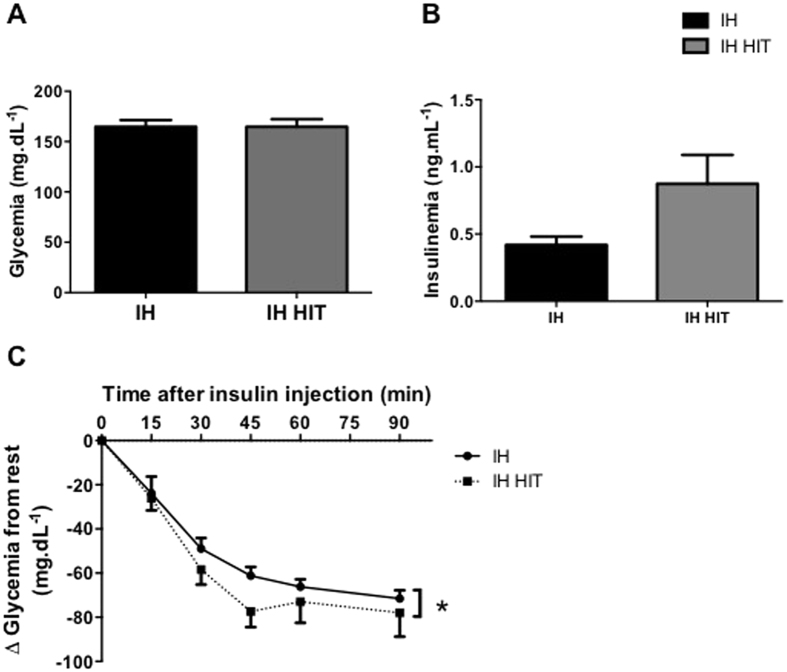
Effects of high intensity training on intermittent hypoxia induced insulin resistance. Mice fasted glycemia (**A**), insulinemia (**B**) and Insulin Tolerance Test (ITT) (**C**) after High Intensity Training. Blood glucose or plasma insulin was measured before intraperitoneally injection of insulin (T0). Data are presented as mean ± SEM. (*p < 0.05; n = 9–10 per group).

High intensity training did not alter fasting glycemia or fasting insulinemia compared to intermittent hypoxia alone (Fig. 5A,B).

Glycemia measured for 90 minutes after the insulin injection decreased more after IH HIT compared to IH exposition alone, suggesting a decrease in insulin resistance (Fig. 5C; F = 3.94; P = 0.05).

### Effects of high training intensity during intermittent hypoxia exposure on insulin and autophagy activation signaling pathways

**Figure 6:**
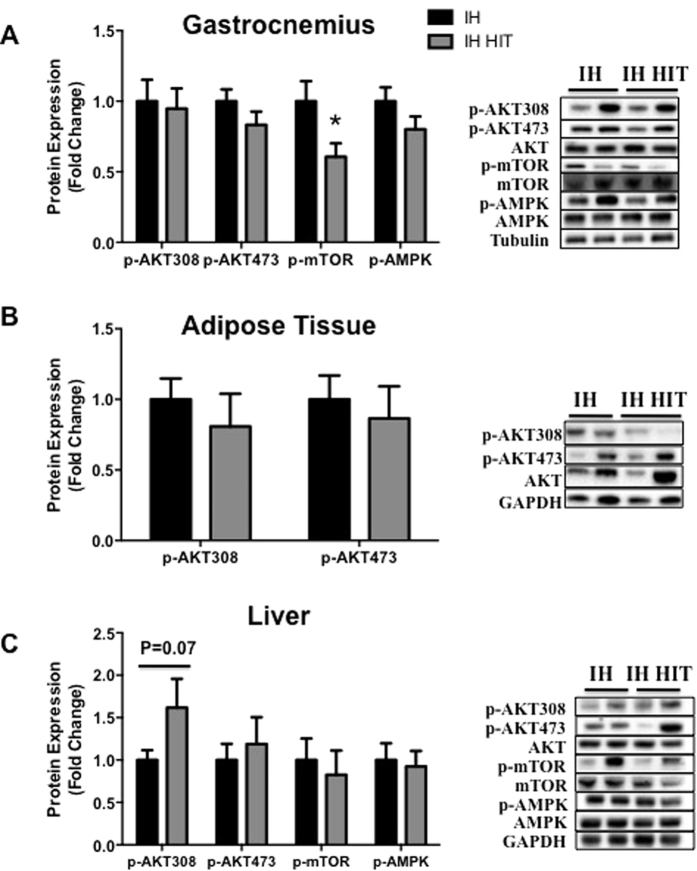
Effects of high training intensity during intermittent hypoxia exposition on insulin and autophagy activation signaling pathways in muscle, adipose tissue and liver. Quantification of phosphorylation of AKT (Thr308 and Ser473), mTOR (Ser2448) and AMPK (Th172) protein expression and representative immunoblot images in (**A**) gastrocnemius muscle, (**B**) adipose tissue and (**C**) liver. AMPK and mTOR were undetectable in adipose tissue. Comparisons were performed after normalization to total protein. Data are presented as mean ± SEM. *P < 0.05 vs IH (n = 9–10 per group).

In *gastrocnemius*, AKT phosphorylations on serine 308 and on Threonine 473, and on AMPK, were not altered after high intensity training compared to intermittent hypoxia alone (Fig. 6A). Conversely, mTOR phosphorylation was lower in IH HIT compared to HIT (Fig. 6A; 0.6 ± 0.1 fold control value, p < 0.05). In *adipose tissue*, AKT phosphorylations on serine 308 and on Threonine 473 did not differ between IH HIT and IH (Fig. 6B). In *liver*, AKT phosphorylations on serine 308 (p < 0.07) and on threonine 473 were greater in IH HIT but differences did not reach statistical significance (Fig. 6C). In addition, phosphorylations of AMPK and mTOR were similar between IH HIT and IH.

### Effects of high intensity training during intermittent hypoxia on autophagy markers

**Figure 7:**
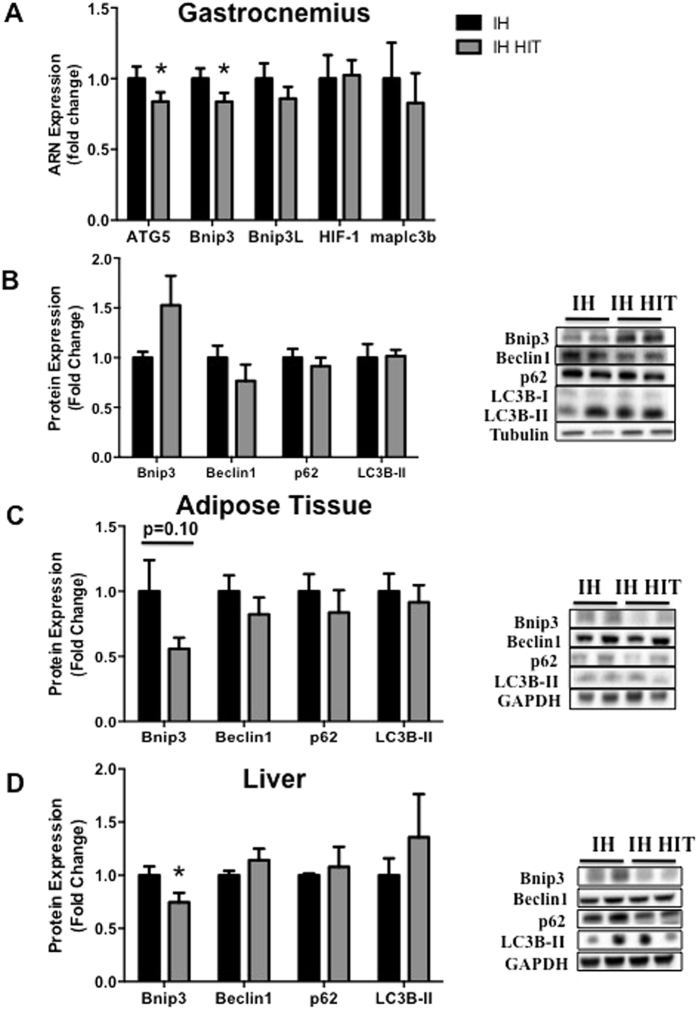
Effects of high intensity training during intermittent hypoxia on autophagy markers in muscle, adipose tissue and liver. ARNm (**A**) and protein (**B**–**D**) analyses were performed on mice that underwent fourteen days of intermittent hypoxia (IH) alone or in association with 3 weeks of high intensity training (IH HIT). Quantification of Bnip3, Beclin1, p62, the ratio LC3-II/LC3-I protein expression and representative immunoblot images in (**B**) gastrocnemius muscle, (**C**) adipose tissue and (**D**) liver. Comparisons were performed after normalization to α-Tubulin or GAPDH. Data are presented as mean ± SEM. *P < 0.05 vs IH (n = 9–10 per group).

In *gastrocnemius*, Bnip3 and ATG5 mRNA relative expressions were lower after IH HIT compared to IH whereas those of Bnip3L, LC3B, HIF-1 were similar (Fig. 7A). Autophagy protein expressions such Beclin 1, p62 and LC3B-II/LC3B-I did not differ between IH and IH HIT (Fig. 7B). Bnip3 protein expression tended to be greater in IH HIT compared to IH but the difference did not reach statistical significance (1.5 ± 0.3 fold control value; p = 0.098; Fig. 7B). In *adipose tissue*, Beclin 1, p62, LC3B-II protein expressions did not differ between IH and IH HIT (Fig. 7C). However, there was a tendency for a lower expression of Bnip3 in IH HIT compared to IH (0.6 ± 0.1 fold control value; p = 0.09; Fig. 7C).

In *liver*, Beclin 1, p62, LC3B-II protein expressions did not differ between IH and IH HIT. However, Bnip3 was significantly lower in IH HIT compared to IH (0.7 ± 0.1 fold control value; p = 0.05; Fig. 7D).

## Discussion

In the present study, we confirmed that intermittent hypoxia induces insulin resistance, characterized by greater fast glycemia, an impaired systemic insulin sensitivity and lower AKT phosphorylation in adipose tissue and liver in our model. When added to IH, HIT induced better systemic insulin sensitivity and a tendency to greater AKT phosphorylation in liver. Thus, our findings suggest that HIT might represent a potential preventive strategy to limit IH-induced IR in OSA patients.

Observational studies have demonstrated that OSA is an independent risk factor for prediabetes and incident diabetes^8^. Both animal and human models of intermittent hypoxia and sleep fragmentation acutely mimicking OSA show evidence of insulin resistance and glucose intolerance^9^. In our study, IH altered the glycemic status and induced insulin resistance. Indeed, fasting glycemia was greater in IH and was not compensated by a hyperinsulinemia. This hyperglycemia might be due to an IR or pancreatic dysfunction associated with insulin secretion perturbation^25^. Also, Shin *et al*. demonstrated that fasting hyperglycemia after four weeks of IH is due to an increase of the sympathetic nervous system, which triggers an increase of baseline hepatic glucose output and hepatic enzymes of the gluconeogenesis (PEPCK)^26^. The insulin tolerance test revealed impaired insulin sensitivity in IH compared to control mice, which confirmed a systemic insulin resistance. Thomas *et al*. have recently shown that hypoxia-inducible factor prolyl hydroxylase 1 (PHD1) deficiency promotes hepatic steatosis and liver-specific insulin resistance in mice^27^. In our study, IR seems also to be tissue dependent. The phosphorylation of protein kinase B (PKB, AKT) is the major signaling pathway modulating glucose metabolism in different tissues^28^. Our results showed that IH induced less phosphorylation of AKT in liver and adipose tissue than in normoxia, which confirms previous results^29^. As opposed to liver and adipose tissue, phosphorylation of AKT (Serine 473 and threonine 308) was greater in muscle after intermittent hypoxia but this could not counteract the systemic insulin resistance. These results contrast with Liyori *et al*.’s observations. These authors attributed the reduced insulin sensitivity to a decrease of glucose disposal and oxidation in the soleus muscle, without modification in the gastrocnemius^6^. In our study, we did not measure AKT phosphorylation in soleus, but gastrocnemius might have partly compensated the insulin resistance in adipose tissue and liver. Nevertheless, this was not sufficient to maintain an adequate glycemic control. Thus, IR induced by IH is systemic and tissue dependent. Of note, this IR occurred despite the greater weight loss largely described in continuous hypoxia conditions^30^, recurrently observed in our lab because of a decrease of food intake. In addition, maximal exercise performance was slightly increased after IH exposition as reported in human beings^31^. The latter could not be explained by a greater hematocrit because this variable was not altered in the present study. However, greater muscle vascularization is a possible explanation because of the well-known effects of IH on HIF1-α and VEGF, one of its targets genes^32^. Furthermore, we cannot exclude a greater maximal cardiac output. However this hypothesis is not consistent with the rather deleterious cardiac effects of IH^24^.

Recent findings highlight the involvement of autophagy in mobilization of diverse cellular energy stores. Thus, autophagy may play an important role in metabolic diseases development. Given the metabolic perturbations associated with OSA or IH, it was reasonable to expect an autophagy perturbation in IR development induced by IH. Few studies have explored autophagy involvement in intermittent hypoxia. Giordano *et al*. showed an activation of autophagy in the diaphragm muscle after 4 days of IH exposure^33^. These authors showed that the diaphragm underwent structural and metabolic changes after short-term IH (4 days exposure), with autophagy-associated atrophy whereas limb muscles were not altered. Moreover, Maeda *et al*. showed the necessity of autophagy integrity to avoid a decrement in myocardium contractile function^20^. In our study, we did not find any alteration in basal autophagy status in muscle, adipose tissue or liver. Autophagy is a dynamic process. A key point that needs to be specified is the difference between measurements that monitor the number or volume of autophagic elements at any stage of the autophagic process versus those that measure flux through the autophagy pathway (the complete process including the amount and rate of cargo sequestered and degraded)^34^. In our study, we did not observe any change in basal autophagy whereas we found a modification of AKT-PI3K signaling pathway involved in autophagy activation. He *et al*. generated mutant mice showing normal levels of basal autophagy but the latter could not be modulated by a stimulus (e.g. exercise)^16^. Other studies should investigate those autophagy dynamic aspects (the autophagy flux) in IH context.

In metabolic syndrome, the beneficial effects of high intensity training on insulin signaling have been shown in muscles and adipose tissue^35^. The second part of our study showed that exercise could improve IH-induced insulin resistance, which is consistent with previously published studies. Indeed, when added to IH, exercise improved systemic insulin sensitivity without changing fasting glycemia or insulinemia. Moreover, HIT tended to improve hepatic phosphorylation of AKT, thus increasing insulin signaling in liver without modification in others tissues. In young obese, exercise improved hepatic insulin sensitivity^36^. Heled *et al*. reported that aerobic exercise training (treadmill) increased hepatic insulin sensitivity due to a greater insulin signaling response and an inhibition of PEPCK activity, a key enzyme of gluconeogenesis, in the hepatocytes of diabetes prone rats^37^. Whether in rodents or humans, exercise improves liver insulin resistance. Moreover, since IH can disturb glucose homeostasis via sympathetic hyperactivity^29^,^38^, we cannot exclude the well-known decrease in sympathetic activity induced by exercise training in the improvement of IR^24^.

In order to find other mechanisms concerning the improvement of insulin sensitivity, we measured the impact of High Intensity Training on autophagy modulation. Indeed, it has been shown that autophagy can mediate chronic exercise-induced metabolic benefits. Furthermore, animal models in which autophagy is perturbed do not adapt to exercise to the same extent. He *et al*. reported that autophagy induction may contribute to the beneficial metabolic effects of exercise^16^. They generated mutant mice (BCL2 phosphorylation mutation) presenting decrease of endurance to exercise and altered glucose metabolism during acute exercise, as well as impaired chronic exercise-mediated protection against high-fat-diet-induced glucose intolerance. These mice showed normal levels of basal autophagy but were deficient in exercise-induced autophagy. In this study, we did not observe a modulation of basal autophagy-related protein as known as the ratio LC3-II/LC3-I and p62 as well as autophagy induction markers. After HIT added to IH, we measured basal autophagy, and more investigation is needed to confirm that stimulus-induced autophagy is not attenuated.

Nevertheless, we observed a decrease of Bnip3 (Bcl-2/adenovirus E1B 19-kDa-interacting protein 3) protein expression in liver and Bnip3 mRNA in muscle after HIT in intermittent hypoxia.

Although Bnip3 is known to be activated through HIF1 and to contribute to mitochondrial autophagy in hypoxia^14^, Bnip3 is also a mitochondrial BH3-only protein that contributes to cell death through activation of the mitochondrial pathway of apoptosis^39^. The decrease of bnip3 could contribute to a decrease of apoptosis in liver, leading to an improvement of the mitochondrial function. Also, Rikka *et al*.^40^ have shown that Bnip3-mediated impairment of mitochondrial respiration induces mitochondrial turnover by activating mitochondrial autophagy^40^. Thus, given the dual role of Bnip3, the decrease of Bnip3 protein expression could be an adaptive response^40^ to reduce apoptosis in liver without modification of autophagy. In diabetes, hepatic insulin resistance is associated with mitochondria alterations and ER stress activation^41^. These two parameters could be interesting to explore to identify mechanisms involved in intermittent hypoxia induced insulin resistance.

In conclusion, our study confirmed the deleterious effects of IH on metabolism and notably insulin resistance without basal autophagy modulation. Moreover, high intensity training improves IH-induced insulin resistance without involvement of basal autophagy. These findings suggest that HIT could represent a preventive strategy to limit IH-induced IR in OSA.

## Methods

### Animals

This investigation conforms to the *Guide for the Care and Use of Laboratory Animals* published by the US National Institutes of Health (NIH Publication no. 85-23, revised 1996).

We used 30 adult males Swiss 129 mice, of 8 weeks old (20–25 g) obtained from Janvier labs (Le Genest-Saint-Isle, France). The study was approved by the University Grenoble Alpes Animal Research Ethic Committee (ComEth) (authorization number: 184_UHTA_U1042_CA_03). The mice were housed (n = 6 or 9 per cage) at the animal care facility of the HP2 laboratory (approval: A38 516 10006), under a 12-hour light-dark cycle with 20–22 °C and allowed free access to standard food and water.

### Experimental design

Mice were first randomly divided into three conditions (n = 10 mice per group): normoxia (N), hypoxia (IH) and hypoxia with high intensity training (IH HIT).

All mice were first accustomed to the treadmill running and aerobic performance tests were evaluated before starting 18 days of high intensity training for the IH HIT group. After 8 days of HIT protocol, all mice were exposed to either normoxia (N) or intermittent hypoxia (IH) for 13 additional days. In each group (N, IH, IH HIT), insulin tolerance test and aerobic performance tests were evaluated before the sacrifice (Fig. 1A).

### Aerobic performance tests and training protocol

Tests and training were achieved on a motorized treadmill (0% grade; Bioseb, Vitrolles, France). Mice were first accustomed to the treadmill running for 4 days before the training and hypoxic protocol (30 min per session). Then, aerobic performance tests were performed before and after hypoxia and training protocol.

#### Aerobic performance tests

MAS (Maximal Aerobic Speed) consisted of a maximal progressive continuous exercise on the motorized treadmill (0% grade). After a 6 min warm-up at 10 cm/s the intensity was increased by 2 cm/s every 3 min until the mice could no longer keep the pace. MAS is the speed corresponding to the last entire stage completed by the animal.

##### Endurance at 75% MAS

Aerobic endurance is defined in this experiment as the running time to exhaustion at 75% of MAS. After a 6 minutes warm-up at 10 cm/min, the speed was increased to reach an intensity of 75% MAS. Exercise was stopped when mice were unable to run.

#### High-intensity aerobic training protocol

HIT consists in 2 bouts of 24 min exercise, interspaced by 30 min recovery. The treadmill speed was set at 10 cm/s for the first step and corresponds to 6 min warm-up (50% of MAS), followed by six steps of 3 min progressive intensity to reach respectively 65, 70, 75, 80, 85 and 90% of MAS.

### Intermittent hypoxia protocol

Animals were exposed daily to 8 hours of intermittent hypoxia (IH) or normoxia (N) during their daytime sleep period, for 16 days. The IH stimulus was performed using a specifically designed device, as previously described (2). It consisted in 1-min cycles alternating 30 s of hypoxia (5% inspired oxygen fraction (FiO_2_) and 30 s of normoxia (21% FiO_2_). FiO_2_ was monitored throughout the experiment with a gas analyzer (ML206, ADInstruments, Oxford, United Kingdom). Normoxic mice were exposed to air streams to reproduce equivalent levels of noise and turbulences related to gas circulation than IH, without hypoxia.

### Insulin tolerance test (ITT)

At the 17^th^ day of the protocol (9 days oh IH), we used the ITT to assess global insulin sensitivity. Mice were fasted for 5 h then weighed before blood was collected from the tail tip for glycemia, insulin rate and hematocrit measurement (T0). Intraperitoneal injection of insulin (0.5 UI.kg^−1^ body weight; Novorapid, Novo Nordisk A/S, Bagsvaerd, Danemark) was made and followed by further blood glucose measurement at 15, 30, 45, 60 and 90 min after the injection.

#### Glycemia

Blood glucose was measured before insulin injection (T0) by blood sampling (≈5 μL) using the glucometer OneTouch^®^ Ultra^®^ (LifeScan France, Issy-Les-Moulineaux, France).

#### Plasma Insulin

Blood samples were collected before the ITT (T0) in heparinized capillaries (≈50 μL). Capillaries (Brand, Finland) were then centrifuged (13000 rpm, 7 min, 21 °C) in order to separate the plasma and the formed elements of blood allowing to measure hematocrit. Plasmas were then collected for blood insulin rate measures. Blood insulin level of each mouse was subsequently measured using a Mouse Insulin ELISA Kit 96-well plate (EMD Millipore, St Charles, Missouri, USA).

#### Hematocrit measurement

Hematocrit was assessed as a ratio of blood cells on the total blood volume.

### Tissue sampling

At the end of the exposure, intraperitoneal injection of insulin (0.5 UI.kg^−1^ body weight; Novorapid, Novo Nordisk A/S, Bagsvaerd, Danemark) was made 10 min before mice were euthanized by cervical dislocation. Liver, epididymal adipose tissue, gastrocnemius, soleus and plantaris muscles were immediately removed and frozen in liquid nitrogen for further analysis (western blot and qPCR analysis).

### Western-blot analysis

Frozen livers, adipose tissues and gastrocnemius muscles were homogenized (Precellys 24, 6500 rpm, 3 × 20 s–5 s; Bertin Technology, Montigny le Bretonneux, France) in order to extract total proteins (Sample lysis buffer: 5 mM EDTA, 1 mM Na_3_VO_4_, 20 mM NaF, 1 mM DTT, proteases inhibitor cocktail). Protein concentration was calculated using a BCA assay (Pierce^®^ BCA Protein, Thermo Scientific, Rockford, USA). Depending on proteins analyzed, 30 to 40 μg of protein were separated by SDS polyacrylamide gels (8–15%) and transferred to polyvinylidene difluoride (PVDF) membranes. Next, membranes were blocked with 5% non-fat milk or bovine serum albumin (BSA) in Tris-buffered saline (TBS) with Tween 20 (0.1%). Then, membranes were incubated overnight at 4 °C with primary antibodies in TBS-Tween 20–5% BSA or non-fat milk (Supplementary Table S1). The following day, membranes were incubated for 1 h at room temperature with horseradish peroxidase-conjugated appropriate anti-IgG (1/5000, Santa Cruz Biotechnology, Heidelberg, Germany). Enhanced chemiluminescence was performed with the Western-Blot ECL substrate (Clarity, Bio-Rad, Marnes-la Coquette, France) according to the manufacturer’s instructions and video acquisition (chemidoc-xrs-system, Bio-Rad, Marnes-la Coquette, France). The relative amount of protein was quantified by densitometry (Image Lab, Bio-Rad, Marnes-la Coquette, France) and expressed as the ratio of loading control. Phospho-proteins were expressed relative to total proteins and not phospho-proteins were expressed relative to GAPDH or tubulin. Finally, protein expressions of different groups were expressed relative to the control groups (N for the evaluation of IH effects or IH when evaluating the effects of IH HIT) normalized at 1.

### Quantitative real-time RT-PCR analysis

Total RNA was collected from *gastrocnemius* muscle using the Total RNA isolation kit (Tri-reagent, Ambion, Austin, TX) followed by purification using the RNeasy Fibrous Tissue Mini Kit (Qiagen). Complementary DNA was generated from 200 ng of RNA using the Eurogentec Core Kit and the C-1000 Thermal Cycler (Bio-Rad). The selected forward and reverse primer sequences are listed in Supplementary Table S2. Real-time quantitative polymerase chain reaction (qPCR) was performed in a 20-μL final volume and optimized concentrations for each primer using the SYBR Master Mix dTTP Blue (Eurogentec). Fluorescence intensity was recorded using a CFX96 Real-Time PCR Detection System (Bio-Rad). Data were analyzed using the ΔΔ*C*_t_ method of analysis^42^. Reference genes (GAPDH, B-Actin and α-tubulin) were used to normalize the expression levels of genes of interest.

### Statistical analysis

Statistics were performed using GraphPad Prism 6 Software (San Diego, California, USA). Data are expressed as mean ± SEM. T-test or unparametric test (Mann & Whitney) were used for two-groups comparisons. For ITT, differences between groups over time were determined by two-way ANOVA, with repetition on the last factor. A p value < 0.05 was considered statistically significant.

## Additional Information

**How to cite this article:** Pauly, M. *et al*. High intensity aerobic exercise training improves chronic intermittent hypoxia-induced insulin resistance without basal autophagy modulation. *Sci. Rep.* **7**, 43663; doi: 10.1038/srep43663 (2017).

**Publisher's note:** Springer Nature remains neutral with regard to jurisdictional claims in published maps and institutional affiliations.

## References

[b1] Dempsey J. A., Veasey S. C., Morgan B. J. & O’Donnell C. P. Pathophysiology of sleep apnea. Physiol. Rev. 90, 47–112 (2010). 20086074 10.1152/physrev.00043.2008 PMC3970937

[b2] Kendzerska T., Gershon A. S., Hawker G., Tomlinson G. & Leung R. S. Obstructive sleep apnea and incident diabetes. A historical cohort study. Am. J. Respir. Crit. Care Med. 190, 218–225 (2014). 24897551 10.1164/rccm.201312-2209OC

[b3] Seicean S. . Sleep-disordered breathing and impaired glucose metabolism in normal-weight and overweight/obese individuals: the Sleep Heart Health Study. Diabetes Care 31, 1001–1006 (2008). 18268072 10.2337/dc07-2003

[b4] Marshall N. S. . Is sleep apnea an independent risk factor for prevalent and incident diabetes in the Busselton Health Study? J. Clin. Sleep Med. JCSM Off. Publ. Am. Acad. Sleep Med. 5, 15–20 (2009). PMC2637161 19317376

[b5] Baron A. D., Brechtel G., Wallace P. & Edelman S. V. Rates and tissue sites of non-insulin- and insulin-mediated glucose uptake in humans. Am. J. Physiol. 255, E769–774 (1988). 3059816 10.1152/ajpendo.1988.255.6.E769

[b6] Iiyori N. . Intermittent hypoxia causes insulin resistance in lean mice independent of autonomic activity. Am. J. Respir. Crit. Care Med. 175, 851–857 (2007). 17272786 10.1164/rccm.200610-1527OC PMC1899294

[b7] Baguet J.-P., Barone-Rochette G., Tamisier R., Levy P. & Pépin J.-L. Mechanisms of cardiac dysfunction in obstructive sleep apnea. Nat. Rev. Cardiol. 9, 679–688 (2012). 23007221 10.1038/nrcardio.2012.141

[b8] Pamidi S. & Tasali E. Obstructive sleep apnea and type 2 diabetes: is there a link? Front. Neurol. 3, 126 (2012). 23015803 10.3389/fneur.2012.00126 PMC3449487

[b9] Pamidi S. . Eight Hours of Nightly Continuous Positive Airway Pressure Treatment of Obstructive Sleep Apnea Improves Glucose Metabolism in Patients with Prediabetes. A Randomized Controlled Trial. Am. J. Respir. Crit. Care Med. 192, 96–105 (2015). 25897569 10.1164/rccm.201408-1564OC PMC4511421

[b10] Yamada E. & Singh R. Mapping autophagy on to your metabolic radar. Diabetes 61, 272–280 (2012). 22275084 10.2337/db11-1199 PMC3266408

[b11] He C. & Klionsky D. J. Regulation Mechanisms and Signaling Pathways of Autophagy. Annu. Rev. Genet. 43, 67–93 (2009). 19653858 10.1146/annurev-genet-102808-114910 PMC2831538

[b12] Chang C.-Y. & Huang W.-P. Atg19 Mediates a Dual Interaction Cargo Sorting Mechanism in Selective Autophagy. Mol. Biol. Cell 18, 919–929 (2007). 17192412 10.1091/mbc.E06-08-0683 PMC1805099

[b13] Alessi D. R. . Mechanism of activation of protein kinase B by insulin and IGF-1. EMBO J. 15, 6541–6551 (1996). 8978681 PMC452479

[b14] Zhang H. . Mitochondrial autophagy is an HIF-1-dependent adaptive metabolic response to hypoxia. J. Biol. Chem. 283, 10892–10903 (2008). 18281291 10.1074/jbc.M800102200 PMC2447655

[b15] Vainshtein A. & Hood D. A. The regulation of autophagy during exercise in skeletal muscle. J. Appl. Physiol. 664–673 (2016). 26679612 10.1152/japplphysiol.00550.2015 PMC4796178

[b16] He C. . Exercise-induced BCL2-regulated autophagy is required for muscle glucose homeostasis. Nature 481, 511–515 (2012). 22258505 10.1038/nature10758 PMC3518436

[b17] Fritzen A. M. . Regulation of autophagy in human skeletal muscle: effects of exercise, exercise training and insulin stimulation. J. Physiol. 594, 745–761 (2016). 26614120 10.1113/JP271405 PMC5341711

[b18] Yang L., Li P., Fu S., Calay E. S. & Hotamisligil G. S. Defective hepatic autophagy in obesity promotes ER stress and causes insulin resistance. Cell Metab. 11, 467–478 (2010). 20519119 10.1016/j.cmet.2010.04.005 PMC2881480

[b19] Singh R. . Autophagy regulates adipose mass and differentiation in mice. J. Clin. Invest. 119, 3329–3339 (2009). 19855132 10.1172/JCI39228 PMC2769174

[b20] Maeda H. . Intermittent-hypoxia induced autophagy attenuates contractile dysfunction and myocardial injury in rat heart. Biochim. Biophys. Acta 1832, 1159–1166 (2013). 23499993 10.1016/j.bbadis.2013.02.014

[b21] O’Hagan C., De Vito G. & Boreham C. A. G. Exercise prescription in the treatment of type 2 diabetes mellitus: current practices, existing guidelines and future directions. Sports Med. Auckl. NZ 43, 39–49 (2013). 10.1007/s40279-012-0004-y 23315755

[b22] Cocks M. & Wagenmakers A. J. M. The effect of different training modes on skeletal muscle microvascular density and endothelial enzymes controlling NO availability. J. Physiol. 594, 2245–2257 (2016). 25809076 10.1113/JP270329 PMC4933120

[b23] Jelleyman C. . The effects of high-intensity interval training on glucose regulation and insulin resistance: a meta-analysis. Obes. Rev. Off. J. Int. Assoc. Study Obes. 16, 942–961 (2015). 10.1111/obr.12317 26481101

[b24] Bourdier G. . High-intensity training reduces intermittent hypoxia-induced ER stress and myocardial infarct size. Am. J. Physiol. Heart Circ. Physiol. 310, H279–289 (2016). 26566725 10.1152/ajpheart.00448.2015

[b25] Drager L. F., Jun J. C. & Polotsky V. Y. Metabolic consequences of intermittent hypoxia: relevance to obstructive sleep apnea. Best Pract. Res. Clin. Endocrinol. Metab. 24, 843–851 (2010). 21112030 10.1016/j.beem.2010.08.011 PMC3011976

[b26] Shin M.-K. . Carotid body denervation prevents fasting hyperglycemia during chronic intermittent hypoxia. J. Appl. Physiol. 117, 765–776 (2014). 25103977 10.1152/japplphysiol.01133.2013 PMC4187050

[b27] Thomas A. . Hypoxia-inducible factor prolyl hydroxylase 1 (PHD1) deficiency promotes hepatic steatosis and liver-specific insulin resistance in mice. Sci. Rep. 6, 24618 (2016). 27094951 10.1038/srep24618 PMC4837354

[b28] Garofalo R. S. . Severe diabetes, age-dependent loss of adipose tissue, and mild growth deficiency in mice lacking Akt2/PKB beta. J. Clin. Invest. 112, 197–208 (2003). 12843127 10.1172/JCI16885 PMC164287

[b29] Gileles-Hillel A., Kheirandish-Gozal L. & Gozal D. Biological plausibility linking sleep apnoea and metabolic dysfunction. Nat. Rev. Endocrinol. 12, 290–298 (2016). 26939978 10.1038/nrendo.2016.22

[b30] de Theije C. C., Langen R. C. J., Lamers W. H., Schols A. M. W. J. & Köhler S. E. Distinct responses of protein turnover regulatory pathways in hypoxia- and semistarvation-induced muscle atrophy. Am. J. Physiol. Lung Cell. Mol. Physiol. 305, L82–91 (2013). 23624791 10.1152/ajplung.00354.2012

[b31] Burtscher M. . Intermittent hypoxia increases exercise tolerance in elderly men with and without coronary artery disease. Int. J. Cardiol. 96, 247–254 (2004). 15262041 10.1016/j.ijcard.2003.07.021

[b32] Semenza G. L. Regulation of oxygen homeostasis by hypoxia-inducible factor 1. 97–106 (2009). 10.1152/physiol.00045.2008 19364912

[b33] Giordano C., Lemaire C., Li T., Kimoff R. J. & Petrof B. J. Autophagy-associated atrophy and metabolic remodeling of the mouse diaphragm after short-term intermittent hypoxia. PloS One 10, e0131068 (2015). 26107816 10.1371/journal.pone.0131068 PMC4480857

[b34] Klionsky D. J. . Guidelines for the use and interpretation of assays for monitoring autophagy (3rd edition). Autophagy 12, 1–222 (2016). 26799652 10.1080/15548627.2015.1100356 PMC4835977

[b35] Tjønna A. E. . Aerobic interval training versus continuous moderate exercise as a treatment for the metabolic syndrome: a pilot study. Circulation 118, 346–354 (2008). 18606913 10.1161/CIRCULATIONAHA.108.772822 PMC2777731

[b36] Van Der Heijden G.-J. . Strength exercise improves muscle mass and hepatic insulin sensitivity in obese youth. Med. Sci. Sports Exerc. 42, 1973–1980 (2010). 20351587 10.1249/MSS.0b013e3181df16d9 PMC2944907

[b37] Heled Y. . Physical exercise enhances hepatic insulin signaling and inhibits phosphoenolpyruvate carboxykinase activity in diabetes-prone Psammomys obesus. Metabolism. 53, 836–841 (2004). 15254873 10.1016/j.metabol.2004.02.001

[b38] Fletcher E. C. Invited Review: Physiological consequences of intermittent hypoxia: systemic blood pressure. J. Appl. Physiol. 90, 1600–1605 (2001). 11247966 10.1152/jappl.2001.90.4.1600

[b39] Gustafsson Å. B. Bnip3 as a Dual Regulator of Mitochondrial Turnover and Cell Death in the Myocardium. Pediatr. Cardiol. 32, 267–274 (2011). 21210091 10.1007/s00246-010-9876-5 PMC3051075

[b40] Rikka S. . Bnip3 impairs mitochondrial bioenergetics and stimulates mitochondrial turnover. Cell Death Differ. 18, 721–731 (2011). 21278801 10.1038/cdd.2010.146 PMC3058880

[b41] Rieusset J. Contribution of mitochondria and endoplasmic reticulum dysfunction in insulin resistance: Distinct or interrelated roles? Diabetes Metab. 41, 358–368 (2015). 25797073 10.1016/j.diabet.2015.02.006

[b42] Pfaffl M. W. A new mathematical model for relative quantification in real-time RT-PCR. Nucleic Acids Res. 29, e45 (2001). 11328886 10.1093/nar/29.9.e45 PMC55695

